# Supporting homecare workers to provide palliative and end-of-life care: A scoping review of interventions and their implementation

**DOI:** 10.1177/26323524261467434

**Published:** 2026-08-04

**Authors:** Lesley E. Williamson, Annika Dhawan, Fatima Mujtabah, Ai Qi Katrina Yeung, Monica Leverton, Katherine E. Sleeman, Abigail Burgess, Clare Ellis-Smith, Catherine J. Evans

**Affiliations:** 1 Cicely Saunders Institute of Palliative Care, Policy and Rehabilitation, 4616 King’s College London, London, UK; 2 Health and Social Care Workforce Research Unit, 388593 King’s College London, London, UK; 3 Sussex Community NHS Foundation Trust, Brighton General Hospital, Brighton, UK

**Keywords:** palliative care, end of life care, homecare, domiciliary care, home health aide, implementation

## Abstract

**Background:**

Social homecare workers provide care to enable people to live as independently as possible in their own homes. They play a central role in supporting palliative and end-of-life care but often encounter challenges and can feel disempowered.

**Objectives:**

We aimed to scope the evidence on interventions to support homecare workers’ provision of palliative and end-of-life care, and the contextual factors that influence their implementation.

**Eligibility Criteria:**

Papers were included if they reported primary research of any design, evaluating any intervention supporting homecare workers to provide palliative and end-of-life care.

**Sources of Evidence:**

We systematically searched four bibliographic databases and supplemented this with reference chaining and grey literature searching.

**Charting Methods:**

Using a qualitative content analysis, we deductively mapped data against the four contextual domains of the Practical Robust Implementation and Sustainability Model.

**Results:**

We found 13 papers reporting interventions to support homecare workers to provide palliative and end-of-life care, of which only seven described contextual factors that influence their implementation. The most common intervention type was training, and most cited contextual factors related to homecare workers’ perspectives of the intervention. Characteristics relating to homecare workers’ schedules and agency staff turnover were also described as influencing implementation of interventions. However, few reported the influence of the perspectives or characteristics of managers, people receiving homecare, and families. Only two of the seven papers mentioned the influence of the external environment or infrastructure to promote implementation and sustainability of interventions.

**Conclusions:**

The scarcity of evidence on interventions and their implementation is a missed opportunity to support the homecare sector’s response to increasing demand for community-based care and reduced hospital deaths. Better understanding of how the workforce can be supported to provide palliative and end-of-life care, in a sustainable way, would help facilitate high-quality care at home for people approaching the end of life.

## Plain language summary

**What is the problem?** Homecare workers provide support to people in their own homes, which can involve caring for people with life-limiting conditions and those close to the end of their life. However, homecare workers report not having enough support or training to do this. There are different ways to support homecare workers to provide end-of-life care, but we do not know what these are or how they can be applied in practice.

**What did we aim to do?** We aimed to find evidence about different ways to support homecare workers to provide end-of-life care, and how best to apply the support in practice.

**What did we do?** We searched for published research articles and unpublished reports on different websites to find evidence on ways to support homecare workers to provide end-of-life care. Within this evidence, we looked at what factors might influence how to apply support for homecare workers into real-life care.

**What did we find?** We found 13 articles that looked at ways to support homecare workers to provide care to people with life-limiting conditions and those close to the end of life. Of these papers, only seven looked at how best to apply the support in practice. Factors that influenced how to apply the support included homecare workers’ views on the support provided, and different features of homecare agencies (for example, staff turnover). There was little evidence found about the influence of the wider care sector (such as private market forces).

**What does this mean?** There is little evidence on what support there is for homecare workers to provide care for people with high levels of need nearing the end of life, and how we should apply this support in practice. We need more research in this area to better prepare homecare workers and the social care sector for increased demand.

## Introduction

Social homecare, also called domiciliary care, involves the provision of care and support to enable people to live as independently as possible in their own homes.^1^ Homecare workers are skilled, although often unregulated, workers supporting personal care (e.g. washing, dressing and toileting), as well as domestic tasks (e.g. meal preparation, cleaning), and promoting social and emotional wellbeing, helping to mitigate unnecessary transfers to hospital and residential care.^2–5^ Social homecare can be provided on a short- or long-term basis, supporting reablement and independence,^2,6^ and can play an essential role in the delivery of palliative and end-of-life care (PEOLC) for those who wish to die at home.^7^ Specifically, they make significant contribution to the holistic management of people with life-limiting conditions, including providing personal care, emotional support to individuals and their families, and escalating concerns to other practitioners.^8^

On the backdrop of an ageing population and political agendas to ‘age in place’ and die at home,^2^ homecare is the frontline provision of social care, and is currently estimated to support around 350,000 older people in England.^2^ As the complexity of care continues to advance, with more people living and dying with multiple comorbidities,^9^ and complex conditions like dementia,^10^ it is projected that 57% more older adults will need homecare by 2038 compared to 2018.^11^ This places greater demand on the homecare workforce, which is already largely untrained, overstretched and undersupported.^12^ Homecare workers are often required to provide care in challenging circumstances with limited information,^13,14^ specifically relating to individual care needs and end-of-life preferences,^15,16^ and can experience challenges when negotiating expectations of any family.^17,18^ They can also experience role ambiguity and isolation,^13,19^ often working within fragmented communication systems, with minimal access to support from peers and healthcare professionals.^14,19^ They experience challenges working interprofessionally;^20,21^ often sitting outside multidisciplinary home-based PEOLC teams,^21^ marginalised by the wider health and social care team,^14^ and perceived as one of the lowest statuses in care work and wider society.^22^ As such, homecare workers can experience significant emotional strain to provide end-of-life care for people,^13,18^ and can feel disempowered to advocate for them.^17^

Homecare workers can benefit from peer and supervisory support,^18,23^ access to support from healthcare practitioners,^24^ and general and condition-specific training,^25^ as advocated in current policy.^26^ However, the homecare sector is characterised by significant workload buden^27^ and fragmented time,^28^ exacerbated by high staff turnover,^25^time-and-task payment,^2^ fiscal austerity measures,^29^ and chronic political neglect.^26^ Little is known about how these contextual factors influence the uptake of supportive interventions into routine homecare.^26^ However, understanding this is fundamentally important to changing practice.^30,31^ Given the unique challenges faced by the homecare sector, careful consideration of *how* interventions are implemented in practice is particularly important.^25^

### Aim and objectives

We aimed to identify the available evidence on factors that may influence the implementation of interventions to support homecare workers to provide PEOLC. To achieve this aim, we had the following objectives:

1. Identify the different interventions intended to support homecare workers to provide PEOLC.
2. Identify the contextual factors that influence the implementation of these interventions.

## Methods

Given the broad scope of our review question, and the potential breadth of evidence sources, we identified a scoping review as the most appropriate method to identify factors and knowledge gaps, as well as more broadly examine how research has been conducted in this area.^32,33^ Our approach was informed by the Arksey and O’Malley framework,^34^ and Joanna Briggs Institute (JBI) methodology.^35^ We used the Preferred Reporting Items for Systematic reviews and Meta-Analyses extension for Scoping Reviews (PRISMA-ScR) Checklist to ensure transparency and standardisation of reporting (supplementary file, S1).^36^ The protocol was registered on OSF Registries (10.17605/OSF.IO/HT8PR). Given the iterative nature of scoping reviews,^32,35^ we made amendments to the protocol following initial registration to improve the comprehensiveness and construct validity of the review. These amendments are recorded in the protocol registration.

### Identifying relevant studies

#### Eligibility criteria

Full eligibility criteria are in Table 1. Given the variation in service structure across countries, there is no shared international term used to describe homecare workers. Therefore, for this review, we defined homecare workers as unregulated social care workers who provide support with personal and domestic care in a person’s own home. We excluded studies that referred to trained care workers whose core role involves performing clinical duties or are supervised by healthcare professionals. We accepted any type of intervention that aimed to support homecare workers to provide PEOLC. This excluded service interventions as implementing new service models or teams to enhance the quality of care would likely have broader contextual factors than interventions to support staff. We included studies that explicitly mentioned palliative and/or end-of-life care, given its broad definition as an approach that prioritises quality of life among people affected by life-limiting conditions,^37^ including those who are in the last year of their life.^38^ We did not specify the client population since social homecare is not condition-specific, there is significant prognostic uncertainty of some conditions, and a life-limiting diagnosis does not in itself warrant end-of-life care by homecare workers. We accepted any year of publication to ensure a comprehensive scoping of evidence and because we had no clear justification to select a specific year limitation.^39^ Similarly, we did not limit by country, though only included papers that were written in English as a pragmatic decision.

**Table 1.** Eligibility criteria.

​	Inclusion	Exclusion
Sample	Homecare workers (any proportion of sample)	Other staff groups, e.g. community/lay health workers, nursing assistants, healthcare assistants, mid-level health workers, residential support workers, and care workers in other residential settings
Phenomenon of interest	Any type of intervention that aimed to support homecare workers to provide PEOLC.	Care interventions (e.g. healthcare input)
		Service interventions (e.g. new palliative care teams or service models)
Design	Any primary qualitative, quantitative or mixed methods design	Systematic reviews or other secondary research syntheses
Evaluation	No limitation	No limitation
Research type	Any original research, including conference abstracts, and letters presenting original findings	Opinion pieces, commentaries, protocols; not original research

#### Information sources and search strategy

Four bibliographic databases, Medline, Scopus, CINAHL and PsycINFO, were systematically searched from September to December 2025. A search strategy for Medline was developed and piloted before adapting the syntax and subject headings for the other databases (supplementary file, S2). This included internationally used search terms for homecare workers (e.g. home health aide). Grey literature was sought from the following sources: Social Care Online, Social Science Research Network, Age UK, Marie Curie, the National Centre for Social Research, the Nuffield Trust, the Care Quality Commission, Homecare Voices, and National Institute for Health and Care Excellence.

### Study selection

#### Study screening and selection

Papers were screened and selected from bibliographic databases using Covidence. Duplicate papers were removed automatically after import, and manually during screening. Screening of titles and abstracts was over-inclusive to exclude clearly irrelevant studies (LEW). All full-text papers were double-screened against the eligibility criteria (LEW, AD, FM, AQKY), with discrepancies resolved through discussion. We supplemented this with screening of primary studies included in relevant evidence syntheses, forward and backward reference chaining (AD, FM, LEW), and grey literature searching (AQKY, FM, LEW).

### Extracting and charting the data

#### Data charting and collating

Data were extracted and double checked using a bespoke template in Microsoft Excel (LW, AD, FM). This included data on study characteristics (country, aim, population and sample, study design, method and analysis), intervention type and contextual factors affecting implementation of interventions. We used a qualitative content analysis as a descriptive approach to analysis to identify characteristics of a specific issue,^40^ deductively mapping evidence to the core contextual domains of the Practical Robust Implementation and Sustainability Model (PRISM).^41^ This method of analysis aligns with JBI methodology for scoping reviews.^40^ PRISM is a conceptual framework to help identify factors needed to implement and sustain an intervention.^42^ Its core contextual domains are: (i) multilevel partner perspectives on the intervention and (ii) multilevel partner characteristics (e.g. at recipient, homecare worker, manager and agency levels); (iii) the external environment (e.g. policies, market forces, regulatory environment and community resources); and (iv) implementation and sustainability infrastructure (e.g. support structure to facilitate adoption, implementation and sustainability).^43^

As a determinants framework, PRISM supports identifying and defining multilevel contextual factors,^44^ facilitates knowledge translation,^41^ can be used across all stages of implementation projects,^42^ and is increasingly used to frame the current state of a field.^45^ While there are other, more comprehensive frameworks that help to assess multilevel contextual factors (e.g. Consolidated Framework for Implementation Research^46^; CFIR), PRISM is more pragmatic to guide implementation planning and differs by including examination of resources and processes to support implementation and sustained delivery.^42^ We considered this helpful to support future implementation efforts in the heterogenous homecare setting in which resources and processes differ. Therefore, it was used to guide the scoping of available evidence and mapping the gaps. Mapping against the core contextual domains was completed independently (AD, FM, AQKY, LEW), with discrepancies resolved by discussion. In the absence of any specific requirement, we did not conduct quality appraisal of included papers, in line with JBI recommendations.^47^

## Results

### Selection of sources of evidence

**Figure 1.**
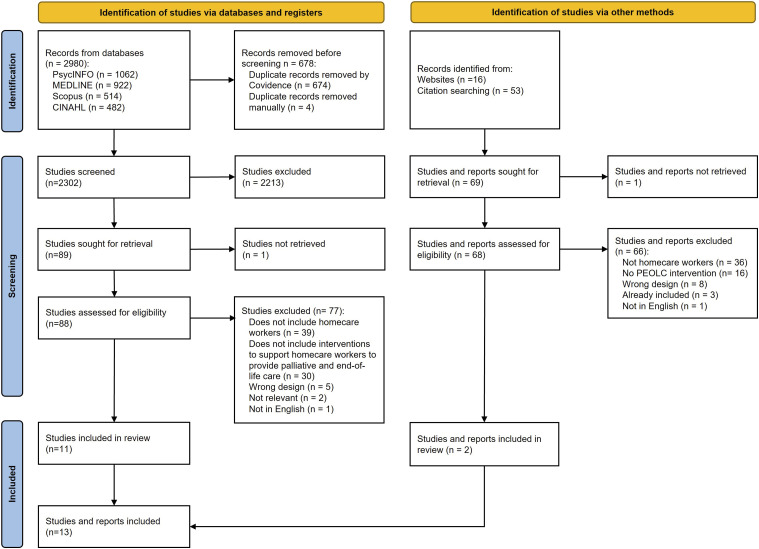
Prisma flow chart.

After de-duplication of imported papers, 2,302 titles and abstracts were screened, 89 were selected for full-text review and 11 studies were included. We included one additional peer-reviewed paper from reference chaining and one unpublished report from grey literature searching and follow-up email correspondence with the author. See Figure 1 for the stages of study selection.

### Characteristics of sources of evidence

The number of studies evaluating interventions to support homecare workers to provide PEOLC have gradually increased across a 25-year period, more so within the last 20 years. There were four studies each from the United Kingdom (UK),^48–51^ United States (US),^52–55^ and Japan,^56–59^ and one study from Sweden.^60^ There were 2,200 different participants represented across the 13 studies, including homecare workers and managers, health and social care professionals, administrators, and others involved in the provision of PEOLC for general adult populations,^49–53,56,57^ and specifically older adults,^55^ or people with dementia,^58,59^ cancer,^60^ or frailty.^48,54^ Most studies comprised multi- or mixed-methods^48–51,53,56,59^; four used quantitative mono-methods,^52,54,57,60^ and two used qualitative mono-methods.^55,58^ Three studies comprised service evaluations,^48–50^ of which one was an unpublished report.^48^ Three studies were randomised control trials,^56,57,59^ two of which were reporting the same trial with the same sample.^56,57^ Table 2 summarises the study characteristics (full characteristics are in supplementary file, S3).

**Table 2.** Summary of study characteristics, according to papers that report and do not report contextual factors influencing implementation.

First author	Country	Design	Key population characteristics	Participants	People receiving homecare	Intervention
*Contextual factors reported*						
Bavetta (2024)^48^	UK	Service evaluation (multi-method)	Domiciliary carers	65 carers	People with severe and advancing frailty	Mixed:
						-6-month training programme delivered by hospice Frailty Lead
						- care coordinator support sessions
						-24-hour advice line
Ferrell (2002)^53^	US	Pre–post evaluation (multi-method)	Homecare nurses, licensed vocational nurses and home health aides in Los Angeles, Orange, San Bernardino, and Riverside	153 staff (125 nurses and 28 home health aides)	People who are terminally ill and their families	HOPE Program (Home Care Outreach for Palliative Care Education): a 5-module palliative care curriculum delivered over six months, inferred delivery by interdisciplinary team of investigators and consultants.
Griffith (2024)^51^	UK	Pre-post evaluation (mixed)	Domiciliary care staff working with people at the end of life	210 staff (186 unregistered care staff)	People receiving end-of-life care	3-day end-of-life care training course devised and delivered by education leads in three collaborating hospices.
Manson (2020)^50^	UK	Service evaluation (mixed)	Home care workers from one domiciliary care agency that delivered care interventions to the community in a large city	25 care staff	People receiving PEOLC	The ECHO programme: 12 x 90mins education sessions, one per month, led and supported by hospice nurses trained in the ECHO methodology.
Nakanishi (2018)^59^	Japan	A cluster-randomised clinical trial (mixed)	Long-term home care providers of the Tokyo Metropolitan Government.	46 staff (7 nurses, 37 care managers, 2 care workers)	People with dementia	Behaviour Analytics & Support Enhancement (BASE) programme,
						2-day training course delivered by unknown facilitators:
						- Baseline assessment
						- Action plan and reassessment
						- Core principle was based on a palliative care approach
Nouri (2020)^54^	USA	Survey (quant)	Patients enrolled in a home-based geriatrics program at the University of California, San Francisco (UCSF).	11 people receiving homecare and their paid carers	Frail older adults with serious illness	“SA-Toolkit”:
						- Script to introduce Symptom Assessment (SA) Tools
						- Guidelines on the next actionable step for the caregiver.
Odierna (2018)^55^	USA	Focus groups (qual)	Workers affiliated to the In-Home Supportive Services (IHSS).	50 participants (20 administrators, 9 case managers, 13 carers, and 8 people receiving homecare.	Older adults	Hypothetical IHSS symptom assessment programme.
*No contextual factors reported*						
Brighton (2018)^49^	UK	Service evaluation (multi-method)	Multidisciplinary health and social care professionals in community settings in South and North West London.	889 health and social care professionals (n=30 carers)	People with serious and life-threatening illness and their families	‘Difficult Conversations’ multidisciplinary, half-day interactive workshop inferred delivery by experienced PEOLC clinicians.
Ferrell (1998)^52^	US	Before and after pilot study (quant)	Homecare professionals (nurses, licensed vocational nurses and home health aides) working in home health agencies in LA.	52 staff (76% nurses, 11% health home aides, 13% other professionals)	People who are terminally ill and their families	HOPE Program (Home Care Outreach for Palliative Care Education): a 5-module palliative care curriculum, inferred delivery by interdisciplinary team of investigators and consultants.
Fujita (2025)^56^	Japan	A cluster-randomised clinical trial (multi-method)	Homecare nurses, care managers, and head care workers in a city with a population of 1.5 million in Japan.	291 (64 nurses, 129 managers, and 98 head care workers).	People who are terminally ill	Interprofessional education programme, delivered by the research team.
Fukui (2019)^57^	Japan	A cluster-randomised clinical trial (quant)	Homecare nurses, care managers, and head care workers in a city with a population of 1.5 million in Japan.	291 (64 nurses, 129 managers, and 98 head care workers).	People receiving end-of-life care in the community.	Interprofessional education programme, delivered by the research team.
Hirakawa (2020)^58^	Japan	Case conference analysis (qual)	Physicians, nurses, care workers providing community end-of-life care in Tohoku, Tokai, Kansai, Shikoku, and Kyushu.	136 staff (11 physicians, 35 nurses, and 90 care workers)	People receiving end-of-life care (focused on people with dementia)	Case conferences discussing refusal of hospital admission, passive euthanasia, and emergency transport.
Thulesius (2002)^60^	Sweden	One-group pretest-post-test design (quant)	Home care staff caring for people at the end-of-life across two rural parts of Sweden.	272 homecare staff providing caring tasks	People living in rural areas with different health conditions, including cancer at the end of life	1yr education project using learner-centred approaches, aiming to develop local guidelines for end-of-life care; headed by a chief nurse and led by a physician with long working experience in advanced palliative home care.

### Summarising and reporting of results

#### Interventions

Of the 13 papers evaluating interventions to support homecare workers to provide PEOLC, ten papers reported educational interventions. Most were standalone training programmes,^49–53,56,57,60^ with resources,^52,53,56,57^ while two studies included training as a component of a wider intervention; one including action planning and assessments,^59^ and the other including care coordinator support sessions and a 24hr hospice advice line.^48^ Training programmes were mostly developed and delivered by healthcare professionals with PEOLC expertise,^48–51,60^ the research team,^52,53,56,57^ or a mix,^56,57^ and ranged from a half-day workshop,^59^ to monthly training sessions over twelve months.^50^ Most training sessions had mixed learners, although three were targeted at homecare workers specifically.^48,50,60^ The sessions focused on condition-specific symptoms,^59^ general palliative care knowledge and skills,^50–53,60^ and specific skills such as communication,^49^ and interprofessional education.^56,57^ The remaining papers reported on a case conference intervention, bringing together different professionals to discuss three palliative care cases,^58^ and hypothetical and piloted symptom assessment toolkits,^54,55^ assessing symptom severity, with guidelines and recommended actions.^54^

#### Contextual factors

Only seven of the 13 included papers described contextual factors that influenced the implementation of interventions.^48,50,51,53–55,59^ The most frequently reported factors influencing implementation were perspectives on the intervention (n=7). Four studies reported the influence of multilevel partner characteristics,^48,50,55,59^ two papers reported the influence of the external environment,^59^ and three reported the influence of implementation and sustainability infrastructure.^48,51,55^ None of the papers were analysed or informed by implementation science (though one intervention was informed by behaviour change theory^54^). The contextual findings are described below (breakdown in supplementary file, S4).

##### Multilevel partner perspectives

All seven papers reported perspectives on the intervention as contextual factors influencing its implementation. These were almost exclusively homecare workers’ perspectives. Generally, interventions were positively perceived by homecare workers as a formal recognition of their work and the need for support of homecare workers,^48,50,54^ and as potentially improving job satisfaction.^55^ However, there were also reported concerns about interventions hypothetically increasing the burden of work,^55^ with specific features of timing and simplicity of interventions being important,^54^ access to resources,^53,54^ and presumed peer support.^55^ Several interventions involved engaging with others, which was well received. Homecare workers appreciated training delivered by clinicians,^48,51^ the opportunity to engage in person,^48,51^ with other homecare workers and other professionals,^48,50,51^ facilitating understanding of specific conditions,^59^ discussion of complex cases,^48,50^ and awareness of and collaboration with providers in the wider health and social care system,^48,50,51^ as well as helping to develop a sense of community by sharing experience and reducing isolation.^50,51^ Interventions involving other professionals also appeared to reduce isolation among homecare workers by providing them with a sense of the bigger picture of PEOLC.^53^ However, homecare workers also welcomed interventions that were personally meaningful,^48,51,53^ and relevant to the scope of their role.^48,50^

While most papers included other actors as participants,^53–55,59^ only two of these reported the perspectives of people receiving homecare,^54,55^ and two reported care managers.^55,59^ One of these papers, exploring views on a hypothetical symptom checklist, also reported the perspectives of homecare administrators.^55^ This study and the other study implementing a symptom checklist demonstrated that people receiving homecare welcome the intervention as a means of accessing support from medical professionals,^55^ and helping them to feel cared for.^54^ However, case managers cautioned about the potential for misunderstanding among people receiving homecare about the purpose of interventions (e.g. fears of overmedicalisation or being admitted to a care home), highlighting the potential importance of careful messaging.^55^

##### Multilevel partner characteristics

The four papers that reported partner characteristics focused primarily on homecare worker and agency characteristics, with minimal reference to characteristics of people receiving homecare. No papers described the influence of manager characteristics on implementation. It was noted in one paper that engagement with homecare agencies as a hospice was initially challenging,^48^ while others noted how the workload and work schedules of homecare workers influenced implementation by reducing their opportunity to engage with the intervention.^50^ This was exacerbated in some agencies by high staff turnover, resulting in significant staff shortage and new staff often being younger and inexperienced,^50,55^ while others had very experienced homecare workers with an appetite to learn.^48^ Developing efficient workflows and procedures was thus considered useful to aid implementation,^50,55^ with incentives to engagement.^48^

Other partner characteristics that could influence implementation included the burden experienced by homecare workers and their varied working knowledge of palliative care,^55^ and different conditions and specific symptoms of people receiving homecare (e.g. neuropsychiatric symptoms in dementia).^59^ For the hypothetical symptom checklist, health literacy and culture were also assumed to be important characteristics among people receiving homecare that influence implementation.^55^

##### External environment

The influence of the external environment on implementing interventions was minimally described; only one paper identified how the siloed nature of homecare agencies prohibited inter-agency working that was required for implementation, and how insurance systems restricted what care homecare workers could deliver.^59^ A second paper referred to the benefits of aligning the intervention with sector regulatory governance requirements.^51^

##### Implementation and sustainability infrastructure

Three studies described initiatives to facilitate implementation and sustainability of interventions. These included leveraging established relationships and agency procedures, such as those used for identifying and escalating concerns,^55^ employing a dedicated training lead,^48^ using champions to cascade learning,^51^ and securing dedicated funding for intervention longevity.^51^

## Discussion

This scoping review found 13 papers reporting on interventions to support homecare workers to provide PEOLC, with only seven of these reporting contextual factors that influenced the implementation of the intervention. The most common intervention type was education and training. The most frequently reported contextual factors were perspectives on the intervention that influenced its implementation, which were mostly homecare worker perspectives, and homecare worker and agency characteristics. The influence of infrastructure to promote implementation and sustainability and the external environment were relatively poorly reported.

We found that multilevel partner perspectives and characteristics were mostly of homecare workers. The evidence on homecare workers’ views and characteristics suggests the need for an educational component of an implementation plan, considering the differences in experience between homecare workers, as well as the benefits of embedding opportunities to engage with other homecare workers and professionals. This is underlined by the preponderance of educational interventions identified in this review, and is corroborated elsewhere by challenges of homeworkers providing PEOLC with minimal training,^20^ differences in educational experience and levels of expertise,^25,61^ and working in isolation,^18,20,25^ excluded from interdisciplinary working.^13,25,62^ It also underscores how interventions place the burden of work on homecare workers to upskill than on other practitioners to more effectively include homecare workers in the delivery of PEOLC, such as through training to better understand, recognise and value the homecare worker role.^21^

We found relatively few papers reported perspectives of homecare managers. Homecare manager characteristics and perspectives may also influence implementation of interventions to support homecare workers provide PEOLC, as wider literature shows how managers’ attitudes towards interventions influence staff engagement,^63,64^ and play a critical role in mitigating infrastructural barriers to implementation.^27,31^ Similarly, we found only two papers included perspectives of people receiving homecare on interventions, reflecting the small number in wider implementation literature.^65^ We found no evidence on the influence of family perspectives, yet wider evidence confirms that family play an essential role in the care of relatives dying at home,^66^ and homecare workers can experience challenges in negotiating family expectations as people receiving homecare approach end-of-life.^17^ Based on these findings, we suggest future research explores how homecare manager, homecare recipient and family perspectives influence implementation, and to consider meaningfully integrating the perspectives of all partners through co-design of PEOLC complex interventions, as advocated elsewhere.^67^

We found little evidence exploring the impact of the external environment on implementing interventions. This is unsurprising when considering wider implementation literature,^65^ but still remarkable given the known pressures within the sector concerning persistent staff vacancies,^25^ and the influence of ‘time-and-task’ payment,^2^ ‘fragmented time’,^28^ market forces,^68^ fiscal austerity measures,^29^ homecare stigma and powerlessness,^22^ public pessismism,^26,69^ and long-term policy failure.^26^ The absence of these factors in this review calls for more research into their influence on implementing interventions that support staff to provide PEOLC, since PEOLC relies heavily on multidisciplinary working,^70^ and continuity,^71^ in the context of clinical complexity and uncertainty,^72^ and widespread misconception.^73^

We found little evidence on infrastructural support for implementation and sustainability. This included the appointment of a dedicated facilitator,^48^ and the influence of champions, which is a highly cited facilitator of implementation.^63,74,75^ Drawing on implementation literature, other facilitators could include adopter training, protocols, and a toolkit to aid implementation.^41,76^ Since infrastructure can influence homecare worker motivation, presenteeism, and the quality of homecare,^62^ as well as capacity building in home-based palliative care,^77^ and issues of high staff turnover are prominent within this heterogenous sector,^25^ we suggest greater focus is afforded to infrastructural support that serve to optimise implementation and sustainability of interventions in homecare settings.

### Implications for future implementation research

While there is limited evidence to guide implementing interventions to support homecare workers to provide PEOLC, the evidence available suggests that implementation requires buy-in from homecare workers and people receiving homecare, and an embedded educational component, tailored to different levels of experience, with opportunities for homecare workers to share experiences, liaise with other professionals, and be recognised for their contributions to the care system and the challenging circumstances in which they work. Plans should also consider aligning interventions with regulatory governance requirements and include champions or dedicated staff to help sustain implementation.

Future research should seek to better understand how implementation is influenced by manager, people receiving homecare and, where possible, family characteristics and perspectives on the intervention, within the context of cultural diversity and sector influences such as service fragmentation, market forces and workforce shortage. Implementation science models or frameworks can support these research efforts by prompting consideration of multilevel factors.^78,79^

### Strengths and limitations

This review is novel in its scoping of evidence on implementing interventions to help homecare workers to provide PEOLC. In doing so, it has identified a significant evidence gap and thus offers original contribution to priority setting in PEOLC research. The review is also significant, given the pressured state of social care as overstretched and underfunded in many countries,^80^ and political agenda to reduce hospital deaths,^81^ reiterating the urgency of implementation research to understand how interventions can best be implemented and sustained to support homecare workers to provide PEOLC. Using an implementation science framework contributes to the robustness of the review findings.^82^ We selected the contextual domains of PRISM to guide analysis. As this is less prescriptive and comprehensive than other implementation science frameworks, such as CFIR, we have not conducted an in-depth evaluation of contextual factors. However, we considered this fitting for the purpose of a scoping review, and the heterogenous nature of homecare. Furthermore, as a pragmatic framework, we have collated evidence to directly inform implementation planning. While there is a risk of bias in mapping the evidence against the four PRISM contextual domains, we minimised this by double checking all extracted and mapped data, as well as double screening all full-text papers, thus increasing the rigour of this review.

The review is further strengthened by our tight definition of homecare workers. However, we acknowledge there is no single internationally accepted definition of, or job title for, social homecare workers, and the nature of homecare is evolving to ‘proxy-healthcare’ with delegated roles.^19^ While we were able to confirm definitions and corroborate the eligibility of papers within the team and with international colleagues and internet searching, it is possible that we may have inadvertently excluded some papers. Similarly, given the broad definition of palliative care, we selected studies that explicitly referenced PEOLC to optimise construct validity, and did not limit to specific terminal conditions. However, we appreciate that in doing so we may have overlooked papers such as those exploring peer support or emotional resilience among homecare workers,^83,84^ or those seeking to enhance competence in dementia care,^27^ which may have offered relevant implementation insights. While we did not include service interventions such as new home-based palliative care services, we acknowledge that they may have indirectly supported homecare workers to provide PEOLC. We also chose not to include outcomes of interventions, as this was not needed to meet our aim of identifying factors that influence implementation. As homecare is a particularly diverse and isolated context for care delivery that is currently understudied, it is of benefit to better understand the factors affecting implementation. However, future research is needed synthesise and compare intervention effectiveness.

The studies in this review were limited to those from high-income countries. This may reflect the greater political appetite for PEOLC in high- compared to low- and middle-income countries,^85^ and other contextual issues,^86^ but it may also reflect the language limitation of our search. While it was a pragmatic decision to limit the search to studies written only in English, we acknowledge that this may have introduced bias and omitted other relevant international evidence. We also acknowledge that the papers included did not aim to evaluate implementation but instead evaluated interventions that had been implemented. However, we did not limit the search to implementation studies, anticipating scarcity of evidence, and still did not find any implementation studies aiming to investigate contextual factors. Therefore, we uphold our finding that there is a gap in the evidence on implementation of interventions to support homecare workers to provide PEOLC.

### Conclusions

This scoping review shows that there has been slow accumulation of evidence on interventions over the past twenty years, most of which involve training and education, but there is comparatively little to inform their implementation. This evidence gap is a missed opportunity to support the sector’s response to the forthcoming increase in the number of older adults likely to be relying on homecare, and highlights a policy blind spot in light of the strong political push to reduce deaths in secondary care.

Rather than continue to flag limitations in social care and the unmet needs of its workforce, there is pressing need to advance knowledge on *how* the workforce can be supported to provide high-quality PEOLC, in a sustainable way, helping people who want to die at home to do so with the best care.

## Supplemental material

Supplemental material - Supporting homecare workers to provide palliative and end-of-life care: A scoping review of interventions and their implementation Supplemental material for Supporting homecare workers to provide palliative and end-of-life care: A scoping review of interventions and their implementation by Lesley E. Williamson, Annika Dhawan, Fatima Mujtabah, Ai Qi Katrina Yeung, Monica Leverton, Katherine E. Sleeman, Abigail Burgess, Clare Ellis-Smith, Catherine J. Evans in Palliative Care and Social Practice
