# Supplementary material for: Supporting homecare workers to provide palliative and end-of-life care: A scoping review of interventions and their implementation
Source: Palliat Care Soc Pract. 2026 Aug 4;20:26323524261467434. doi: 10.1177/26323524261467434 (PMC13438218; doi:10.1177/26323524261467434)
Supplement: Supplementary file 1 [file sj-pdf-1-pcr-10.1177_26323524261467434.pdf]

SUPPLEMENTARY FILES

S1: PRISMA-ScR CHECKLIST....................2

S2: SEARCH STRATEGIES....................4

S3: STUDY CHARACTERISTICS AND FINDINGS....................6

S4: CONTEXTUAL FACTORS INFLUENCING IMPLEMENTATION (PRISM DOMAINS)....................12

S1: PRISMA-ScR CHECKLIST

SECTION	ITEM	CHECKLIST ITEM	REPORTED ON PAGE #
**TITLE**			
Title	1	Identify the report as a scoping review.	1
**ABSTRACT**			
Structured summary	2	Provide a structured summary that includes (as applicable): background, objectives, eligibility criteria, sources of evidence, charting methods, results, and conclusions that relate to the review questions and objectives.	2
**INTRODUCTION**			
Rationale	3	Describe the rationale for the review in the context of what is already known. Explain why the review questions/objectives lend themselves to a scoping review approach.	4-5
Objectives	4	Provide an explicit statement of the questions and objectives being addressed with reference to their key elements (e.g., population or participants, concepts, and context) or other relevant key elements used to conceptualize the review questions and/or objectives.	5
**METHODS**			
Protocol and registration	5	Indicate whether a review protocol exists; state if and where it can be accessed (e.g., a Web address); and if available, provide registration information, including the registration number.	5
Eligibility criteria	6	Specify characteristics of the sources of evidence used as eligibility criteria (e.g., years considered, language, and publication status) and provide a rationale.	5-6
Information sources	7	Describe all information sources in the search (e.g., databases with dates of coverage and contact with authors to identify additional sources), as well as the date the most recent search was executed.	6
Search	8	Present the full electronic search strategy for at least 1 database, including any limits used, such that it could be repeated.	6
Selection of sources of evidence	9	State the process for selecting sources of evidence (i.e., screening and eligibility) included in the scoping review.	7
Data charting process	10	Describe the methods of charting data from the included sources of evidence (e.g., calibrated forms or forms that have been tested by the team before their use, and whether data charting was done independently or in duplicate) and any processes for obtaining and confirming data from investigators.	7
Data items	11	List and define all variables for which data were sought and any assumptions and simplifications made.	7
Critical appraisal of individual sources of evidence§	12	If done, provide a rationale for conducting a critical appraisal of included sources of evidence; describe the methods used and how this information was used in any data synthesis (if appropriate).	NA
Synthesis of results	13	Describe the methods of handling and summarizing the data that were charted.	7
**RESULTS**			
Selection of sources of evidence	14	Give numbers of sources of evidence screened, assessed for eligibility, and included in the review, with reasons for exclusions at each stage, ideally using a flow diagram.	7
Characteristics of sources of evidence	15	For each source of evidence, present characteristics for which data were charted and provide the citations.	8-11
Critical appraisal within sources of evidence	16	If done, present data on critical appraisal of included sources of evidence (see item 12).	NA
Results of individual sources of evidence	17	For each included source of evidence, present the relevant data that were charted that relate to the review questions and objectives.	9-11
Synthesis of results	18	Summarize and/or present the charting results as they relate to the review questions and objectives.	12-14
**DISCUSSION**			
Summary of evidence	19	Summarize the main results (including an overview of concepts, themes, and types of evidence available), link to the review questions and objectives, and consider the relevance to key groups.	14-16

SECTION	ITEM	CHECKLIST ITEM	REPORTED ON PAGE #
Limitations	20	Discuss the limitations of the scoping review process.	16-17
Conclusions	21	Provide a general interpretation of the results with respect to the review questions and objectives, as well as potential implications and/or next steps.	17-18
**FUNDING**			
Funding	22	Describe sources of funding for the included sources of evidence, as well as sources of funding for the scoping review. Describe the role of the funders of the scoping review.	19

S2: SEARCH STRATEGIES

MEDLINE(R) ALL 1946 to September 30, 2025	
1	exp home health aide/ or (homecare or domiciliary or "paid care*" or "home care aid*" or "homecare work*" or "personal care*" or "homecare assistan*" or "ancillary staff" or "care assistant" or "care support worker" or "care worker*" or "cna" or "community support worker" or "domiciliary care assistants" or "home helper" or "home-care support worker" or "home-care worker" or "in-home supportive services" or "lpn" or "social carer" or "unqualified aids").mp.
2	exp terminal care/ or exp terminally ill/ or exp palliative care/ or exp attitude to death/ or (“end-of-life” or “end of life” or “dying at home” or dying or “advanc* disease” or life-limiting).mp.
3	(intervention or innovation or train* or educat* or "outcome measure" or tool* or guid* or resource* or supervis* or support or program* or practice or technolog* or digital).mp.
4	1 AND 2 AND 3

APA PsycInfo 1806 to September 2025 Week 5	
1	exp home care/ or exp home care personnel/ or exp home care/ or (homecare or domiciliary or "paid care*" or "home care aid*" or "homecare work*" or "personal care*" or "homecare assistan*" or "ancillary staff" or "care assistant" or "care support worker" or "care worker*" or "cna" or "community support worker" or "domiciliary care assistants" or "home helper" or "home-care support worker" or "home-care worker" or "in-home supportive services" or "lpn" or "social carer" or "unqualified aids").mp.
2	exp Terminally Ill Patients/ or exp Palliative Care/ or exp "Death and Dying"/ or exp death attitudes/ or "end-of-life".mp. or "end of life".mp. or "dying at home".mp. or dying.mp. or "advanc* disease".mp. or "life-limiting".mp.
3	exp intervention/ or (innovation or train* or educat* or "outcome measure" or tool* or guid* or resource* or supervis* or support or program* or practice or technolog* or digital).mp.
4	1 AND 2 AND 3

Scopus 2025 Elsevier B.V.	
1	“home health aide” or homecare or domiciliary or "paid care*" or "home care aid*" or "homecare work*" or "personal care*" or "homecare assistan*" or "ancillary staff" or "care assistant" or "care support worker" or "care worker*" or "cna" or "community support worker" or "domiciliary care assistants" or "home helper" or "home-care support worker" or "home-care worker" or "in-home supportive services" or "lpn" or "social carer" or "unqualified aids"
2	terminal care or palliative care or “end of life” or “dying at home” or dying or “advanc* disease” or life-limiting
3	intervention or innovation or train* or educat* or "outcome measure" or tool* or guid* or resource* or supervis* or support or program* or practice or technolog* or digital
4	1 AND 2 AND 3

CINAHL 2025 EBSCO	
1	(MH "Home Health Aide Service (Saba CCC)") OR (MH "Home Health Aides") OR (MH "Home Health Agencies") OR Homecare or Domiciliary OR paid care* OR personal care* OR homecare assistan* OR Ancillary staff OR Care assistant OR care support worker OR care worker OR CNA OR community support worker OR domiciliary care assistan* OR home helper OR home-care support worker OR home-care worker OR in-home supportive services OR LPN OR social carer OR unqualified aids
2	(MH "Terminal Care") OR (MH "Terminally Ill Patients") OR (MH "Palliative Care") or end-of-life or dying at home or dying or advanc* disease or life-limiting
3	(Intervention or innovation or train* or educat* or "outcome measure" or tool* or guid* or resource* or

	supervis* or support or program* or practice or technolog* or digital)
4	1 AND 2 AND 3

S3: STUDY CHARACTERISTICS AND FINDINGS

First author, date	Country	Aim	Population / sample / included studies	Client group	Study design, method and analysis / synthesis	Intervention description	Training type	Contextual factors?
Bavetta, 2024	UK	To improve domiciliary care providers' confidence and ability to care, demonstrate an increase in referrals from carers to hospice services and reduce incidents of unscheduled emergency care.	*Population:*<br>Domiciliary carers<br><br>*Sample:*<br>65 care workers from four homecare agency partners.	People with severe and advancing frailty.	*Design:*<br>Service evaluation<br><br>*Method:*<br>Online questionnaire (with open and closed questions) and general feedback discussions during training<br><br>*Analysis:*<br>Descriptive.	• Training programme delivered by hospice staff and guided by the NHS/skills for health – 'Frailty: A framework of competencies' and 'End of Life framework Core Skills Education and Training framework' three components of training were produced 'Let's talk frailty' 'Understanding palliative care' and 'Care at the end of Life'. Ran over 6 months.<br>• Regular support sessions were established with agency care coordinators to discuss complex cases, advise and encourage appropriate signposting and referral to hospice or other appropriate services.<br>• All domiciliary care provider partners were offered access to support via the St Clare Hospice nurse-led 24-hour Advice Line if required.	Mixed (training, support sessions, 24-hour advice line)	Yes
Brighton, 2017	UK	To evaluate the self-reported impact of a multidisciplinary half-day interactive workshop, and gain feedback to inform future development and evaluation.	*Population:*<br>Multidisciplinary health and social care professionals: across community settings in South and North West London serving a population of 4.7 million.<br><br>*Sample:*<br>886 health and care professionals: qualified or trainee general practitioners (34%), community nurses and care coordinators (32%), social care professionals (7%), care home staff (6%), advanced practice/specialist nurses (5%), care workers (5%, n=30) and allied health professionals (3%).	People with serious and life-threatening illness and their families.	*Design:*<br>Service evaluation<br><br>*Method:*<br>Pre–post questionnaires (quantitative self-ratings and qualitative feedback via free-text comments to rate their confidence, knowledge and skills in conducting difficult conversations with patients and carers; and questions about the likelihood the workshop would improve their practice, and whether they would recommend it to a colleague)<br><br>*Analysis:*<br>T-tests to compare scores between matched and unmatched questionnaires at baseline and post-workshop. Qualitative feedback was analysed using inductive content analysis.	'Difficult Conversations' multidisciplinary, half-day interactive workshop developed by experienced palliative and end of life care (EoLC) clinicians. Involved an interactive seminar, video examples and group work covering the principles of breaking bad news, and introducing the 'SCARS' (Setting, Communicate with kindness, Ask, Respond and reflect and Summary and plan) communication framework.	Training	No

Ferrell, 1998	US	To describe and pilot test a training programme named HOPE (Home care Outreach for Palliative care Education), designed to improve the knowledge and skills of home care professionals.	*Population:* Homecare professionals (nurses, licensed vocational nurses and home health aides) working in home health agencies in LA.<br><br>*Sample:* 52 staff (76% nurses, 11% health home aides, and 13% as other professionals) from two homecare agencies in the LA area.	People who are terminally ill and their families.	*Design:*<br>Before and after pilot study.<br><br>*Method:*<br>Self-rated effectiveness and agency effectiveness before first session and two months later.<br><br>*Analysis:*<br>Descriptive statistics and t-tests.	HOPE Program (Home Care Outreach for Palliative Care Education) — developed by researchers at the City of Hope National Medical Centre.<br><br>5-module palliative care curriculum; 1) Explore the importance of EOL care issues in home care. 2) Discuss the principles of pain management at the EOL. 3) Describe symptom management in EOL care. 4) List communication strategies with dying patients and family members in home care settings. 5) Discuss nursing interventions regarding the death event in home care settings.	Training	No
Ferrell, 2002	US	To improve end-of-life care agencies through implementation of a palliative care curriculum.	*Population:*<br>Homecare professionals (nurses, licensed vocational nurses and home health aides) providing care to terminally ill patients and families in community home settings working in home health agencies in the counties of Los Angeles, Orange, San Bernardino, and Riverside.<br><br>Sample:<br>125 nurses (nurses and licensed vocational nurses) and 28 home health aides from five homecare agencies.	People who are terminally ill and their families.	*Design:*<br>Pre–post evaluation of an educational intervention.<br><br>*Method:*<br>Surveys (before and after education), chart audits, case study analyses, and agency evaluation forms. Phases: pre-education observation (2 months), 6-month education phase, and 1-month follow-up. Included: Knowledge Assessment, Individual Evaluation tool (own EOLC effectiveness) and Agency evaluation forum (evaluate HOPE curriculum, completed by agency directors).<br><br>*Analysis:*<br>Quantitative analysis of pre/post test scores (knowledge assessment) and descriptive analysis of participant ratings and open-ended survey responses.	HOPE Program (Home Care Outreach for Palliative Care Education) — developed by researchers at the City of Hope National Medical Centre.<br><br>5-module palliative care curriculum delivered over six months within home care agencies. Modules: 1) Introduction to palliative care and end-of-life issues; 2) Understanding pain and pain management; 3) Understanding end-of-life symptoms and how to manage them; 4) Communication, spirituality, and family caregivers; 5) The signs and symptoms approaching death. Participants received written and video materials and agency resource kits.	Training	Yes
Fujita, 2025	Japan	To verify the understanding and awareness of the elements of an interprofessional education (IPE) programme.	*Population:*<br>Homecare nurses, care managers, and head care workers in a city with a population of 1.5 million in Japan.<br><br>*Sample:*<br>291 (64 homecare nurses, 129 care managers, and 98 head care workers).	People who are terminally ill.	*Design:*<br>A cluster-randomised clinical trial.<br><br>*Method:*<br>Four districts were randomly assigned as education groups, and three districts were randomly assigned as control groups. A questionnaire survey was conducted before the workshop as a baseline (T1) and seven months later (T2) in an education group and a control group. In the education group only, participants were asked for qualitative feedback about the IPE programme immediately after the workshop through a free answer question in the questionnaire.<br><br>*Analysis:*<br>Quantitative data: Two-way repeated measures ANOVA to test for a difference between the education and	The IPE programme:<br><br>• Educational booklet created by research team (including nurses), based on focus groups and literature review.<br>• Educational workshop (lecture, group work session and role play)<br>• Follow-up aimed at (a) understanding own and other professional roles on end-of-life care and (b) mutual respect as a member of a team of different professionals in end-of-life care.<br><br>The IPE program was taught in July 2016.	Training	No

					control groups over time; paired t-test to test simple main effect on time.<br>Qualitative data: Independently coded by three professional respondents; codes were categorised and defined based on similarities and differences. This was conducted with discussion among the authors.			
Fukui, 2019	Japan	To assess the effect of a multidisciplinary end-of-life educational intervention program on confidence in inter-professional collaboration and job satisfaction among health and social care professionals.	*Population:*<br>Homecare nurses, care managers, and head care workers in a city with a population of 1.5 million in Japan.<br><br>*Sample:*<br>291 (64 homecare nurses, 129 care managers, and 98 head care workers).	Patients receiving end-of-life care in the community.	*Design:*<br>A cluster-randomised clinical trial.<br><br>*Method:*<br>Baseline data collected in June 2016 (T1) before the intervention by a questionnaire survey. Experimental group attended two 3-hour programmes in the 7-month study period. Seven months later, in January 2017 (T2), follow-up data were collected. Following the same protocol, the control group received the intervention after T2 (as a wait-list control).<br><br>*Analysis:*<br>T-tests, chi-square tests, or Mann-Whitney U tests to assess comparability between the groups. The effects of the intervention on each measure were assessed using multivariate regression analyses to test for differences between the experimental and the control groups over time, after adjusting for variables that showed p-values < .10 between groups in the univariate analysis.	The IPE programme:<br>• Educational booklet created by research team (including nurses), based on focus groups and literature review.<br>• Educational workshop (lecture, group work session and role play)<br>• Follow-up aimed at (a) understanding own and other professional roles on end-of-life care and (b) mutual respect as a member of a team of different professionals in end-of-life care.<br><br>The IPE program was taught in July 2016.	Training	No
Griffith, 2024	UK	To evaluate a training programme to upskill unregulated domiciliary care agency staff and integrate them into the palliative care teams, supporting registered nurses in caring for end-of-life patients.	*Population:*<br>Domiciliary care staff working with people at the end of life.<br><br>*Sample:*<br>210 staff attended the teaching. They were mostly informal carers, with a few managers, senior team leaders and education leads for their agencies (186 unregistered care staff).	Patients receiving end-of-life care.	*Design:*<br>Mixed-methods evaluation<br><br>*Method:*<br>• Pre-post questionnaire: Self-rated confidence, against specific skills.<br>• Written statement: 2 months after completion, how practice had changed.<br>• Informal interviews: with some agency teams to build on this qualitative data.<br>• Managers asked if call numbers to local hospices seeking advice had increased, and if staff could identify whether unnecessary calls to 999 and 111 had been prevented by the training.<br><br>The follow-up was repeated at 6 months.<br><br>*Analysis:*<br>Descriptive analyses from	3-day end-of-life care training course devised and delivered by education leads in three collaborating hospices, mapped on to the Key Lines of Enquiry for inspections from the Care Quality Commission (independent regulator of health and social care providers).	Training	Yes

					questionnaire data. Thematic analysis of qualitative data.			
Hirakawa, 2020	Japan	To examine the effectiveness and efficiency of interprofessional case conferences on homebased end-of-life care.	*Population:*<br>Physicians, nurses, care workers providing community end-of-life care in 5 regions: Tohoku, Tokai, Kansai, Shikoku, and Kyushu.<br><br>*Sample:*<br>136 healthcare professionals (11 physicians, 35 nurses, and 90 care workers).	People receiving end-of-life care (case conferences about people with dementia).	*Design:*<br>Case conference analysis<br><br>*Method:*<br>Three case conferences involving three cases which entailed the following ethical dilemmas pertaining to home-based end-of-life care: refusal of hospital admission, passive euthanasia, and emergency transport.<br><br>*Analysis:*<br>Qualitative content analysis and Jonsen's four topics approach: medical indication, patient preferences, quality of life (QOL), and contextual features.	Three cases were discussed during the case conferences: refusal of hospital admission, passive euthanasia, and emergency transport.<br><br>Discussions lasted no more than 90 minutes.	Case conference	No
Manson, 2020	UK	To test the acceptability of Project ECHO (extension for community healthcare outcome) to domiciliary care workers as a means of increasing their knowledge of, and confidence in, delivering palliative care, and its effectiveness in reducing isolation by developing a community of practice.	*Population:*<br>Home care workers from one domiciliary care agency that delivered care interventions to the community in a large UK city, including a night roaming service, and frequently delivered palliative and end of life care.<br><br>*Sample:*<br>64 attendances over the 12 education sessions from 25 members of care staff (average 2.24).	Not specified	*Design:*<br>Mixed-methods service evaluation (May 2018 to April 2019).<br><br>Qualitative:<br>- Focus group, to evaluate enjoyment of the programme and understand what could be improved if the programme was run again. Focus group recordings were transcribed by the researcher.<br><br>Quantitative:<br>- Knowledge and confidence self-efficacy questionnaire designed by the ECHO team, issued before starting education sessions, after six sessions and following the last session.<br><br>*Analysis:*<br>- Descriptive statistics.<br>- Inductive, line-by-line coding followed by categorisation was performed. Themes identified using content analysis, and discussed with the team to clarify context<br>- Triangulation to improve credibility.	The ECHO programme: 12 x 90mins education sessions, one per month, led and supported by hospice nurses trained in the ECHO methodology.<br>• Before the 12 sessions started, participants were invited to an event at which gaps in their knowledge were identified, and a curriculum was created based on their needs.<br>• Each session consisted of a short lecture and case study discussion focused on palliative and end of life care topics identified by the participants	Training	Yes
Nakanishi, 2018	Japan	To investigate the effects of the Behaviour Analytics & Support Enhancement (BASE) programme on the attitudes towards dementia care among care managers and	*Population:*<br>Long-term home care providers of the Tokyo Metropolitan Government.<br><br>*Sample:*<br>46 participants received training in August 2016; 24 long-term home care providers, 3 districts. 37 care	People with dementia.	*Design:*<br>Mixed methods (repeated measures)<br><br>Quantitative:<br>- Baseline and follow-up questionnaire survey using Japanese version of the Approaches to Dementia Questionnaire (ADQ) and	Complex intervention - Behaviour Analytics & Support Enhancement (BASE) programme:<br>• 2-day training course, developed via a workshop between a Japanese working group and a Swedish team from the Behavioural and Psychological Symptoms	Mixed (training, assessment, action plan, monthly multi-agency discussion)	Yes

		professional caregivers of home care services.	managers, 7 nurses, and 2 other care workers. 141 clients with dementia registered by participants at baseline.		the Zarit Burden Interview (ZBI).<br><br>Qualitative:<br>- Follow-up meeting to observe and record reflections from participants. After the meeting, each member of the research team reviewed the reflections and identified common issues that were regularly expressed throughout the 3-day workshop.<br><br>*Analysis:*<br>Changes in total scores of the ADQ, SCIDS, and ZBI scales: paired t-test. Effect size calculated with Cohen's d for each outcome measure using the correlation between the two means.	of Dementia (BPSD) Program of Care<br>• Baseline assessment of clients added to web-based tool<br>• Action plan through multi-agency discussions<br>• Ongoing monitoring and reassessment with monthly multi-agency discussion to reassess the behaviour and unmet needs, and revise the action plan for the client.<br><br>1-day follow-up meeting for debriefing		
Nouri, 2020	US	To pilot a Symptom Assessment (SA) Toolkit for caregivers to assess older adults' symptoms at home	*Population:*<br>Patients enrolled in a home-based geriatrics program at the University of California, San Francisco (UCSF), which has 24/7 telephone access to a clinician and the capability of urgently seeing patients when needed, and their paid caregivers.<br><br>*Sample:*<br>A convenience sample of 11 patients and their paid carers.	Frail older adults with serious illness (≥55yrs) excluding people with diagnosis of active drug or alcohol abuse, psychosis, or dementia, or who did not pass a telephone screen for cognitive impairment.	*Design:*<br>Pre- and post-survey design<br><br>*Method:*<br>Pre- and post-surveys administered over phone or in person to patients and caregivers at baseline (time of recruitment) and follow-up (1-2 weeks after using the SA-Toolkit).<br><br>*Analysis:*<br>Descriptive and inferential statistical analyses.	SA-Toolkit:<br>• Script for caregivers to introduce SA<br>• Tools for assessing symptom severity (Symptom Board, Symptom Cards, Faces Scale, Symptom Tracker sheet)<br>• Guidelines on the next actionable step for the caregiver.<br><br>The Toolkit is meant to provide all needed education, and no additional education sessions were provided to the patient or the caregiver. The Social, Cognitive and Behavioural Change theory was used to develop the SA-Toolkit.	Symptom assessment	Yes
Odierna, 2018	US	To explore whether and how to integrate symptom assessment into an IHSS program to identify and manage symptoms in diverse older adults who receive in-home care.	*Population:*<br>Workers affiliated to the In-Home Supportive Services (IHSS) with ≥1 year's affiliation and self-reported proficiency in spoken English<br><br>*Sample:*<br>Convenience sample of 50 people (≥18yrs) in 10 groups; 20 in 4 administrator groups, 9 in 2 case manager groups, 13 in 2 IHSS provider groups, and 8 in 2 consumer groups (≥55yrs).	Older adults (≥55yrs) excluding people with self-reported dementia or psychosis, or did not pass a phone screen for cognitive impairment.	*Design:*<br>Qualitative, Focus groups<br><br>*Method:*<br>10 semi-structured, 90-minute focus groups, with separate groups for administrators, case managers, IHSS providers, and consumers.<br><br>Discussion guides informed by the advisory board, palliative care and aging experts, and previous studies. Topics included potential role of an IHSS symptom assessment program, barriers and facilitators, and logistical factors that could impede or support implementation.<br><br>*Analysis:*<br>Deductive and inductive thematic analysis.	Hypothetical IHSS symptom assessment programme.	Symptom assessment	Yes
Thulesius, 2002	Sweden	To evaluate a 1-year learner-centred educational project in end-of-life care for homecare staff in a rural district of Sweden.	*Population:*<br>Home care staff caring for people at the end-of-life across two rural parts of Sweden.<br><br>*Sample:*	Clients living in rural areas with many types of health problems, including end-of-life care in	*Design:*<br>One-group pretest-post-test design.<br><br>*Method:*<br>A 1-year learner-centred educational project in end-of-life care for home	Education project headed by a chief nurse and led by a physician with long working experience in advanced palliative home care, and trained in learner centred education in Cardiff, Wales. Learner-centred approaches mixing small group work with lectures,	Training	No

			272 homecare staff providing caring tasks.	cancer.	care staff in a rural district of Sweden. Another rural district in the same region served as a control area.<br><br>• Questionnaire: measure attitudes towards different aspects of end-of-life care.<br>• HAD: measure mental wellbeing of staff.<br><br>*Analysis:*<br>Inferential statistics	seminars, and discussions, and with the objective to produce local guidelines for end-of-life care.		

S4: CONTEXTUAL FACTORS INFLUENCING IMPLEMENTATION (PRISM DOMAINS)

First author, date	Perspectives on intervention	Multilevel partner characteristics	External environment	Sustainability and implementation infrastructure
Bavetta, 2024	Delivered at hospice site at the request of the agencies – they felt their staff responded best to face to face learning and the agencies did not have space to deliver training, it was also felt that visiting the hospice site would introduce their people to the hospice to break down barriers to people's engagement and help carers support their service users.<br><br>Care coordinators benefited from the opportunity to review and reflect on difficult situations around complex deaths of clients, instances of difficult transfers of care or unscheduled hospital admissions.<br><br>A general theme in discussion with those attending was how much they appreciated the respect given to them in terms of the calibre and time commitment of educators and the level of knowledge imparted. They stated that training offered previously had been at a low level and did not understand the roles they were being asked to play as domiciliary carers.<br><br>Care providers developed a deeper understanding of the support available to them as well as their clients both from the hospice and local community health services. Hospice staff involved in the teaching sessions expressed deeper respect and understanding of the challenges faced by the care providers and their commitment to providing an excellent service to their clients.<br><br>Training sessions should offer a variety of activities and deliveries to cater for all learners, and encourage carers to share their expertise and experiences.	Care providers attending often bought a wealth of experience and expertise in their roles alongside an enthusiasm to share and learn more. This became particularly apparent as they became aware of the respect that St. Clare's staff have for their role in patient care.<br><br>Ability to engage with clients of care agencies: Recruitment and commitment of care agencies initially proved challenging, of the 14 agencies approached 10 agencies either did not respond to approaches by email and phone follow up or declined engagement at first approach. However, with perseverance and offers of free lunches, 4 agencies eventually took up the offer of free training and support and were recruited as project partners.<br><br>Those attending training had many years of experience in the sector to share and an appetite to learn and improve their knowledge.	Not applicable.	A new Hospice-based Frailty Lead role was created to provide education and training in palliative care to domiciliary care workers.
Ferrell, 2002	Many of the positive comments focused on receiving new resources and expanding their understanding of palliative care, including the 'whole picture' of the dying patient, cultural differences and receiving a resource binder.<br><br>Suggestions for change/improvement included increasing number and frequency of sessions, and more time for more personal experiences.	Not applicable.	Not applicable.	Not applicable.
Griffith, 2024	Attendees commented on the value of receiving the teaching in-person.<br><br>Participants noted that they felt inspired by the teachers and supported when the content of the course became emotive.<br><br>Attendees also commented on the value of learning with colleagues from other agencies and the value of the interactive teaching and shared experiences.<br><br>All attendees did report that they were now calling	Not applicable.	The training was mapped on to the Key Lines of Enquiry (KLOEs) for CQC inspection. The praise from CQC inspectors and regional award given to a care agency following the teaching, further demonstrate improvements in the standard of care given.	Many of the course attendees have progressed to become 'champions' for their organisations in end-of-life care. This ongoing development is supported by the collaboration delivering 'champions' days' provided annually to further the education in end-of-life care,<br><br>The number of attendees who felt able to cascade the training to others in their teams, at the end of each course, was 100%. When followed up at 6 months post course, 87.5% reported that they had already

	their local hospices for help and support and felt empowered to do so, knowing that their calls would be welcomed, rather than previous fears of being treated as a nuisance. This was evidenced by 66% of attendees noting that the course had enabled them to make close relationships with their local hospice.			shared the learning with colleagues.<br><br>The model of this teaching course is sustainable, with ongoing funding from the Better Care Fund.
Manson, 2020	Participants described increased confidence in caring for people approaching the end of life. They reported that the opportunity to discuss cases with others made them feel less isolated in their role.<br><br>Several participants valued the peer support and sense of community within the sessions.<br><br>Participants also stated that the ECHO programme validated their skills and helped them to feel that their work was recognised.<br><br>Participants said they would prefer content that was more relevant to their role as home carers.<br><br>The participants valued having a variety of professionals with whom to discuss their complex cases. One participant discussed the difficulty of contacting professionals about complex cases and said that taking part in the ECHO programme had helped to break down some of those barriers	Participants reported that they worked seven days/nights on and had seven off, and so because the sessions were held monthly it meant that the same staff could attend but also meant that some staff could not attend any.<br><br>The domiciliary care agency involved in this study reported some organisational challenges and staff departures during the time for the sessions, which had resulted in staff shortages<br><br>Evaluation forms were completed directly following each session, however due to workload the participants often had to leave early, which resulted in missing data.	Not applicable.	Not applicable.
Nakanishi, 2018	Several participants reported challenges in setting up a discussion on an action plan among professional caregivers from various organizations.<br><br>The discussion process helped improve the understanding of persons with dementia	Different caregivers had different perspectives on the challenging behaviour of persons with dementia. This could result in difficulties in integrating divergent perspectives.	Some of the suggested action plan components such as massage, activity and music/song were not allowed under the benefit schedule of the public, long-term care insurance scheme.<br><br>Challenges in completing the intervention (i.e. setting up a discussion on an action plan among professional caregivers as they were from various organizations, e.g. one client with dementia received personal care at home from several different care workers and the care manager was not involved in direct caregiving but had to implement the action plan via other caregivers such as nurses and direct care workers). Challenges that the participants raised in the follow-up meeting may imply the challenges in dementia care due to the current long-term care system rather than deficits of the BASE programme.	Not applicable.

Nouri, 2020	Caregivers rated all components of the SA-Toolkit highly because they ‘‘were simple and easy to use.’’<br><br>Caregivers felt supported by the materials: ‘‘Nice to know that people care to help caregivers’’<br><br>All caregivers reported the amount of time it took to assess symptoms using the SA-Toolkit and Symptom Tracker was appropriate.<br><br>Some caregivers also requested more guidance on next steps in symptom management.<br><br>The easy-to-use SA-Toolkit was acceptable, useable, and welcomed by patients and their caregivers.<br><br>Discrepancy in views of whether the information in the SA Toolkit was appropriate for caregiver - areas lacking included personalisation, notes of what the symptoms may be a "bigger warning for" and info to keep track of medication alongside symptoms.	Not applicable.	Not applicable.	Not applicable.
Odierna, 2018	Many consumers welcomed symptom assessment, knowing they would have help getting information back to their medical providers, especially as many are isolated and homebound.<br><br>Administrators and Consumer participants cautioned about potential misunderstanding and cultural perceptions about symptom assessment.<br><br>Training was also thought to increase IHSS providers’ job satisfaction to ‘‘help them feel like they’re doing their job.”<br><br>Need to use scripts, checklists, standard assessment tools – ‘‘To have a list of things each time to remind you to check off.”<br><br>IHSS provider and Consumer were concerned that the programme may burden overworked IHSS-staff.<br><br>Participants also warned that clients might object to overmedicalisation of a program historically focused on activities of daily living and may fear sharing medical information with IHSS providers: ‘‘My client was scared that [family] might put her in a home" (Case Management staff)	Need for careful messaging ‘‘to use terms that drive us toward quality of life’’ and to address health literacy and cultural differences; (Administrator)<br><br>The new people that turn over really fast… our experienced population is moving into retirement, and a new wave is coming in and that has been happening for several years now. The vast majority are very young and very inexperienced, inexperienced in the working world let alone in homecare<br><br>Homecare workers do a demanding job and require more support and higher pay.		Leverage established procedures and relationships, between clients and homecare workers, and case managers and homecare workers.
